# Application of the Delphi Method for Content Validity Analysis of a Questionnaire to Determine the Risk Factors of the Chemsex

**DOI:** 10.3390/healthcare11212905

**Published:** 2023-11-05

**Authors:** Pablo del Pozo-Herce, Antonio Martínez-Sabater, Elena Chover-Sierra, Vicente Gea-Caballero, Pedro José Satústegui-Dordá, Carles Saus-Ortega, Clara Isabel Tejada-Garrido, Mercedes Sánchez-Barba, Jesús Pérez, Raúl Juárez-Vela, Iván Santolalla-Arnedo, Enrique Baca-García

**Affiliations:** 1 Department of Psychiatry, Fundación Jiménez Díaz University Hospital, 28040 Madrid, Spain; pablo.pozo@quironsalud.es (P.d.P.-H.); ebaca@quironsalud.es (E.B.-G.); 2 Instituto de Investigación Sanitaria de la Fundación Jiménez Díaz, 28040 Madrid, Spain; 3 Nursing Care and Education Research Group (GRIECE), GIUV2019-456, Nursing Department, Universitat de Valencia, 46010 Valencia, Spain; antonio.martinez-sabater@uv.es (A.M.-S.); elena.chover@uv.es (E.C.-S.); 4 Care Research Group (INCLIVA), Hospital Clínico Universitario de Valencia, 46010 Valencia, Spain; 5 Internal Medicine, Consorci Hospital University of Valencia, 46014 Valencia, Spain; 6 Research Group Community Health and Care, International University of Valencia, 46002 Valencia, Spain; vagea@universidadviu.com; 7 Faculty of Health Sciences, International University of Valencia, 46002 Valencia, Spain; 8 SAPIENF (B53_23R) Research Group, Faculty of Health Sciences, University of Zaragoza, 50018 Zaragoza, Spain; pjsd@unizar.es; 9 Nursing School La Fe, Adscript Centre, University of Valencia, 46026 Valencia, Spain; saus_car@gva.es; 10 Research Group GREIACC, Health Research Institute La Fe, 46016 Valencia, Spain; 11 Research Group in Care, Faculty of Health Sciences, University of La Rioja, 26006 Logroño, Spain; clara-isabel.tejada@unirioja.es (C.I.T.-G.); ivan.santolalla@unirioja.es (I.S.-A.); 12 Faculty of Medicine, University of Salamanca, 37007 Salamanca, Spain; mersanbar@usal.es (M.S.-B.); jesusperez@usal.es (J.P.); 13 Department of Psychiatry, University of Cambridge, Cambridge CB2 1TN, UK; 14 Prevention and Early Intervention in Mental Health (PRINT), Biomedical Institute of Salamanca, 37008 Salamanca, Spain

**Keywords:** chemsex, content validity, Delphi method, Delphi technique, expert consensus, experts judgment, instrument development, instrument validation, psychometrics, validity

## Abstract

Chemsex is understood as “the intentional use of stimulant drugs to have sex for an extended time among gay, bisexual, and other men who have sex with men”. It is a public health problem because of the increased incidence of cases and because of the consequences on the physical and mental health of those who practice it. Aim: This study aimed to analyze, with the help of the Delphi method, the content validity of a new instrument to assess the risk of behaviors associated with the chemsex phenomenon. Method: First, a bank of items identified from the literature was elaborated. Secondly, 50 experts with knowledge of the chemsex phenomenon at the national level were contacted. A Delphi group was formed with them to carry out two rounds of item evaluation. The linguistic evaluation (comprehension and appropriateness) was assessed using a Likert scale from 1 to 5 for each item. Items that did not reach a mean score of 4 were eliminated. Content assessment was calculated using each item’s content validity index (CVI) and Aiken’s V (VdA). A minimum CVI and VdA value of 0.6 was established to include the items in the questionnaire. Results: A total of 114 items were identified in the literature. In the first round of Delphi evaluation, 36 experts evaluated the items. A total of 58 items were eliminated for obtaining a CVI or VdA of less than 0.6, leaving 56 items. In a second Delphi round, 30 experts re-evaluated the 56 selected items, where 4 items were eliminated for being similar, and 10 items were also eliminated for not being relevant to the topic even though they had values higher than 0.6, leaving the scale finally composed of 52 items. Conclusion: A questionnaire has been designed to assess the risk of behaviors associated with the chemsex phenomenon. The items that make up the questionnaire have shown adequate content and linguistic validity. The Delphi method proved to be a helpful technique for the proposed objective.

## 1. Introduction

Chemsex is understood as “the intentional use of stimulant drugs to have sex for a long period among gay, bisexual, and other men who have sex with men” and is a public health problem because of the increased incidence of cases [1,2] and because of the consequences on the physical and mental health of those who practice it [3,4,5,6]. According to Madrid’s recent 2022–2026 Addictions Plan, care for people who practice chemsex has increased from 50 in 2017 to 351 in 2021, an increase of 602% in the number of people served in recent years [7].

It should also be noted that the increased use of geolocation applications has led to an increase in unprotected sexual encounters, which is associated with increased risk behaviors for sexually transmitted infections (STIs) [4,8]. Previous studies indicate the association of chemsex with the priority consumption of substances such as mephedrone, methamphetamine, cocaine, GHB/GHL [9] and the search for achievement of emotions, pleasurable sensations, and management of negative symptoms [10]. Other studies indicate that individuals with substance use-related problems used in chemsex may have experienced early adverse events and may have an avoidant insecure attachment style. In addition, those who have been diagnosed with HIV may show greater emotional dysregulation and worse self-care patterns. These variables should be routinely assessed in this population [11].

The assessment of mood disorders and addiction linked to the practice of chemsex is of interest given the psychoactive substances used [11]. The practice of chemsex has been linked to increased suicide, sexually transmitted infections (STIs) [12,13,14,15], psychosis [16], and mental problems [9], and decreased adherence to pre-exposure prophylaxis treatment (PrEP) among others [17].

It is of interest to raise awareness of chemsex as a public health problem among gay, bisexual, and other men who have sex with men (GBMSM). Specific identification, education [18] and prevention programs need to be strengthened to reduce the incidence of the most undesirable implications of sexualized drug use (USD) among GBMSM [10].

The literature indicates the importance of self-monitoring for the reduction of harm from chemsex use [19] as well as the development of different programs that allow computer applications [18] and other applications (app) [20,21] to support and inform participants, reduce the negative impacts associated with chemsex and encourage more reasoned participation. On the other hand, the lack of knowledge of professionals in our country [22] regarding a growing problem should be considered.

Thus, studies such as Nagington’s in 2022 indicate that we suggest that medicalized forms of chemsex support could benefit from more rigorous and rapid forms of assessment for problematic chemsex, and also provide infrastructure and training for peer support initiatives. We also suggest that medical services can learn from patients and their peers about support needs that professional services continue to miss and engage in collaborative approaches to practice development [23]. Early detection and knowledge of risk factors can contribute to the reinforcement of accessible, non-judgmental, and well-informed prevention and harm reduction activities to support MSM who engage in slamsex [24]. In addition, an equity-oriented approach should be adopted to facilitate unbiased care opportunities [25].

This phenomenon has raised public health concerns, as it can lead to risky behaviors, such as unsafe sexual practices and an increase in sexually transmitted infections, as well as addictions and mental health problems. Chemsex raises essential questions about the intersection between sexuality, drug use, and health and highlights the need to address this issue comprehensively, providing support and education to those who engage in these practices to minimize the associated risks.

However, to date, the risk factors for chemsex have not been specifically addressed, nor is there currently any instrument available to assess them. All of the above can slow down the detection of these warning signs by the health professionals who work with them, who care for them, and who understand that the quality of life of these people is fundamental. Therefore, it is necessary to know the predictive behaviors to improve their care and holistic well-being. To address and respond to the abovementioned needs, this study aimed to design a questionnaire to detect risk factors for chemsex.

## 2. Materials and Methods

### 2.1. Design

The study carried out was of the instrumental type, which, according to Montero and León (2005), is research that develops tests and devices comprising both the design or adaptation and the study of their psychometric properties. The scale will, therefore, be subject to validation in Spanish.

### 2.2. Item Bank Construction

The questionnaire was developed between July and August 2022. A literature search was conducted, and reference studies related to the research topic were analyzed, verifying the absence of instruments available for the study. The initial version of the questionnaire entitled “*Chem-Sex Inventory*” (CSI) was organized into six sections consisting of 114 items from various validated scales. It is important to note that the scales were not used as such; instead, items were selected from each questionnaire to create the new instrument. All the tools used to develop the “Chem-Sex Inventory” (CSI) questionnaire have been validated in the Spanish environment. The first block related to anxiety consisting of 7 items (from 1–7) (GAD 7) [26]; the second block associated with depression (PHQ-9) [27] consisting of 9 items (from 8–16 items), the third block related to the risk of psycho (PQ-B) [28], Scale of Corrigan [29], and CAPE-15 scale [30], consisting of 21 items (from 17–37), 14 items (from 88–99), and 15 items (from 100–114), respectively, the fourth block related to impulsivity (BIS-11) [31] consisting of 30 items (from 38–67), the fifth block associated with body perception (PHQ-15) [32] composed of 15 items (from 68–82), and finally the sixth block related to suicide risk and consisting of 5 items (from 83–87) (Paykel Scale) [33]. The researchers initially eliminated two items associated with hospital admission from the Corrigan Scale.

Each research committee member, including all the study’s authors, collaborated in developing the new questionnaire, defining its structure and main characteristics, selecting the items, and reviewing them.

### 2.3. Selection of Experts

Fifty experts on the subject were contacted, understanding as experts those professionals who had more than five years of experience in their field and professional trajectory and at least two years of experience in the management of users who practice chemsex, knew about the chemsex phenomenon, were active specialists in their field, and had a direct relationship with users who practice chemsex. The 50 experts comprised 10 LGTBI+ individuals, 10 mental health professionals, 10 emergency and urgent care professionals, 10 primary care professionals, and 10 professionals from infectious disease and sexually transmitted infection (STI) units.

The experts were invited to participate in the study directly by e-mail. Together with the e-mail, a letter of introduction to the survey was sent informing about chemsex and the risk factors associated with the phenomenon and an information sheet describing the characteristics of the study, the objectives of the research, as well as the selection criteria, the confidentiality of the data, and the voluntary nature of the study. The participation of this group of experts was carried out voluntarily, anonymously, and confidentially using a questionnaire through the Microsoft Forms platform. Before disseminating the first survey, the experts identified were asked to accept the Declaration of Consent if they were interested in participating in the study according to the Data Protection Law in force in Spain.

### 2.4. Delphi Method

The conventional Delphi method was used through an iterative process in which experts were consulted in two rounds [34]. Linstone and Turoff (1975) consider that two rounds are sufficient to reach a consensus, allowing adequate reflection on the group’s responses [35].

The rounds were developed through different phases from August 2022 to January 2023. This first phase of construction of a questionnaire on the risk behaviors of the chemsex phenomenon is addressed to the experts, who were asked to evaluate both the relevance and comprehensibility of each of the items using a Likert-type scale between 1 (strongly disagree) and 5 (strongly agree) to clarify the aspects and form of the future questionnaire. A qualitative question on the relevance and clarity of the sections was also added, in addition to criteria of completeness, wording, and structuring of each item. Secondly, the responses of the group of experts were received. Subsequently, a discussion group was held, where suggestions were considered. Finally, the experts’ responses were collected, integrating the pertinent modifications, and the final version of the questionnaire was defined.

Communication with the experts took place from 28 July 2022, when the cover letter for acceptance of participation in the study was disseminated. The first round was broadcast on 4 August 4 2022 and the second round was issued on 4 October 2022, finally closing on 28 January 2023.

#### 2.4.1. Round 1: Content Validity/Linguistic Validity and Loss of Experts Are Evaluated

The first round of consultation was used to evaluate the content validity (appropriateness) and linguistic validity (comprehensibility) of each item. After this first round, the number of items considered in the second round was significantly reduced.

#### 2.4.2. Round 2: Content Validity Assessed

In the second round, the content validity of the items was evaluated (although in some cases there were still some items with adequate content validity but low scores in comprehensibility that were re-evaluated for comprehensibility).

### 2.5. Content Validity Analysis

The content validity of the questionnaire was analyzed by calculating the content validity index and Aiken’s V value for each item. A minimum CVI and Aiken’s V value of 0.6 was established to include the items in the questionnaire, the criterion used to select the items. Based on the experts’ scores, the indicators were calculated. Following the methodology described by Polit and Beck [36], and used by other authors [37,38,39], three indicators of content validity were calculated for each item (CVI, kappa coefficient (k), and Aiken’s V), based on the ratings made by the group of experts, using the following equations:

(a) Content validity index (CVI)

*CVI = number of experts who evaluated the item with 4 or 5 (A)/Total of experts (N)* (1)

(b) =Modified kappa (k)

$$k=\frac{\left(I-CVI\right)-Pc}{1-Pc}$$

In which the I-CVI is the coefficient of internal validity, previously calculated for each item, whereas the Pc (probability of chance agreement) is the probability of chance in accordance between observers and is calculated through the formula:

$$Pc=\left[\frac{\left[N!\right]}{\left[A!\left(N-A\right)!\right]}\right]\times 0.5^{N}$$

(c) Aiken’s V. Its equation, algebraically modified by Penfield and Giacobbi (2004) [40], is:

$$V=\frac{X-l}{k}$$

*X* is the mean of the experts’ ratings, *l* is the lowest possible score, and *k* is the range of possible values of the Likert scale used. For example, if lowest score is 1 and the highest score is 5, then *k* = 5 − 1= 4. Once calculated, Aiken’s V confidence intervals were obtained using the scoring method [41]. To obtain this confidence interval, the following equation was used for the lower limit of the interval:

$$L=\frac{2nkV+z^{2}-z\sqrt{4nkV(1-V)+z^{2}}}{2(nk+z^{2})}$$

And for the upper limit of the interval:

$$U=\frac{2nkV+z^{2}+z\sqrt{4nkV(1-V)+z^{2}}}{2(nk+z^{2})}$$

*L*: lower limit of the interval; *U*: upper limit of the interval; *Z*: value in standard normal distribution; *V*: Aiken’s V calculated by formula 1; *n*: number of experts.

The CVI, modified kappa, and Aiken’s V were calculated with a database created in Excel 2013, using the assessments of the expert group and according to their respective formula.

### 2.6. Comprehensibility Analysis/Linguistic Validation

To obtain the validity of comprehension, the experts were asked to evaluate the degree of understanding of each item in the first round and whether they considered that any should be reformulated. The average score is calculated for such items. Items with scores above 4 were supposed to be of high comprehensibility; those with scores between 3.5 and 4 were supposed to be of medium comprehensibility; and those with scores below 3.5 were considered to be of low comprehensibility. The items that obtained lower scores in the first round and were selected for the second round because of their content validity were reformulated, so their comprehensibility was re-evaluated in the second round.

### 2.7. Ethical Considerations

The study was conducted under the Declaration of Helsinki, and was approved by the Committee of the University of La Rioja with verification code (CSV) (D2R1m2Iu3vLVPdIzGZVnK0h6N558tCyN) for human studies through this link: https://sede.unirioja.es/csv/public/index.xhtml;jsessionid=2D8AB94A16FEB14C923EFD5E63C25AF2-n1.ma_07, accessed on 19 September 2023.

## 3. Results

### 3.1. Characteristics of Experts

Of the 50 experts initially considered, 72% (36 experts) agreed to participate and completed the first round. The second and final round was completed by 30 experts (60%), 70% of whom were men and 30% women (Table 1). Other noteworthy characteristics of the group of experts are the eight mental health professionals, five emergency professionals, six primary care professionals, seven infectious disease professionals, and four LGTBI+ professionals. They came from seven autonomous communities: Aragón (n = 1); Extremadura (n = 2); Canary Islands (n = 2); Catalonia (n = 5); Valencian Community (n = 1); La Rioja (n = 3); Madrid (n = 16). Concerning their profession, 56.66% (n = 17) were nurses, 33.33% (n = 10) were physicians, 6.66% (n = 2) were psychologists, and 3.33% (n = 1) were social workers and sexologists.

### 3.2. Results of the Delphi Method

**Figure 1:**
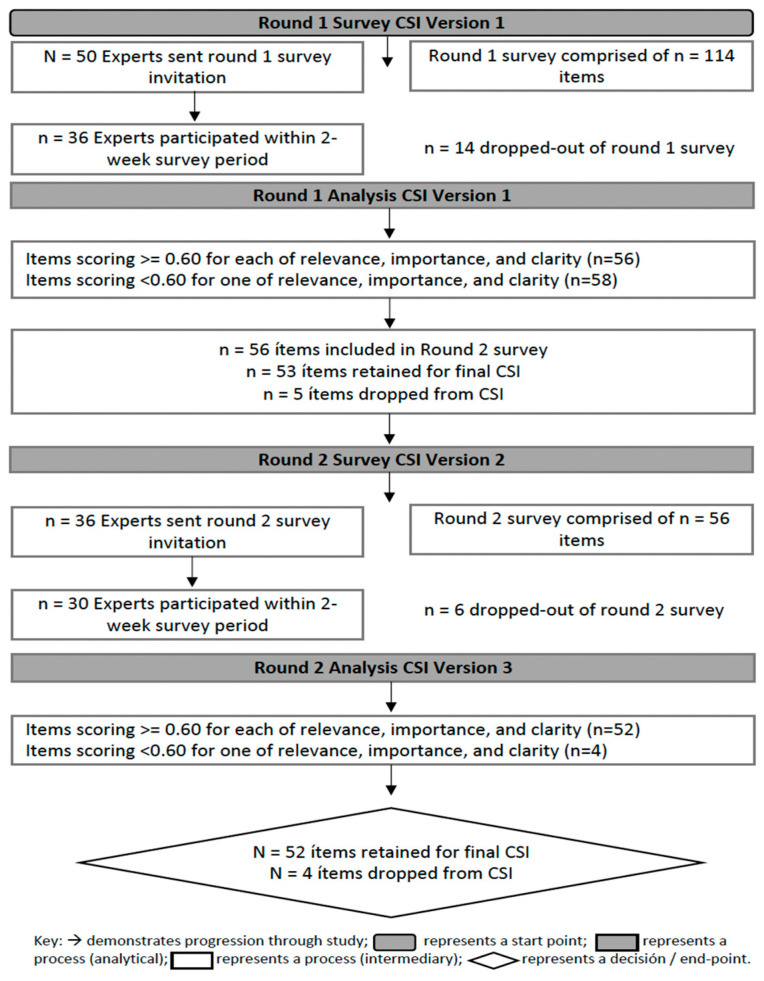
Final summary of the Delphi method. Own elaboration adapted [42].

Figure 1 shows the questionnaire development process and the content validity index (I-CVI) of each of the 114 items from the Delphi rounds. Of these 114 items, 56 (49.1%) had an I-CVI > 0.6 after the first round and 58 items with an I-CVI < 0.6 were eliminated. After the second round, 52 items (92.8%) exceeded the cut-off value, eliminating the remaining 4 items.

### 3.3. Content Validity Analysis

The criterion used to select the items that made up the final questionnaire was that the CVI value or Aiken’s V test score was higher than 0.6. See Table 2.

Thirty-six experts completed the first round. After a review of the first round, those items that had obtained a minimum CVI and VdA value of 0.6 were selected. Of the 114 items that comprised the questionnaire (Appendix A), 61 met both relevance and comprehensibility criteria. Of these 61 items, we proceeded to analyze the experts’ observations for reformulation and understanding of items 43, 44, 63, 65, 65, 73, 91, 92, and 97. The rest of the experts’ suggestions were not considered because they were related to items that did not obtain a value higher than 0.6 and were therefore eliminated from the questionnaire. In addition, the group of experts considered that questions 11 and 81, 16 and 84, 10 and 82, 65 and 67, 9 and 83 (which, in principle, all met the criteria for permanence) were similar, so 5 of these 10 questions were eliminated, leaving 56 items finally selected after the first round.

A total of 30 experts completed the second round out of the 36 who initially completed the first round. After a review of the second round, the same criteria as in the first round were maintained, selecting items with a minimum CVI and VdA value of 0.6. Of the 56 items in the questionnaire, the items that did not obtain a minimum CVI and VdA value of 0.6 were eliminated, leaving 54 items that met the criteria. According to the experts’ observations, questions 11 “Have you had difficulty concentrating when doing everyday things, such as reading the newspaper or watching television?” and 26 “Are you a person who has difficulty concentrating?” were similar, so item 26 was eliminated because the question was less complete. Subsequently, after review by the research group, two similar items were observed, item 55 “Have you ever heard voices when you were alone?” and item 56 “Have you ever heard voices talking to each other when you were alone?”, so the second item was removed. Finally, a questionnaire with 52 items was obtained.

When analyzing the content validity of the 52 items through the CVI and Aiken’s V, the researchers eliminated 10 items for not being relevant to the topic even though they had values above 0.6. The values of CVI and Aiken’s V for each of the items that make up the questionnaire are shown in Table A1 (Appendix A). Analyzing the CVI and Aiken’s V for each item, we see that 97.6% of them reached the value of 0.6 considered acceptable.

Lawshe (1975) suggests that a CVI = 0.29 will be adequate when 40 experts have been used, a CVI =0.51 will suffice with 14 experts, but a CVI of at least 0.99 will be necessary when the number of experts is 7 or less [43]. Although classically a value of 0.70 has been taken for the Aiken’s V cut-off point [44] and 0.8 for the CVI [45] the team set the cut-off point at 0.6, firstly in order not to be so permissive, secondly because there are many reviewers, and thirdly because of the possible further reduction of items with the following phases of the instrument development.

Only the following two items, “Have you ever seen things that other people cannot see?” and “Have you ever felt as if you were under the control of any external force or power?” scored below 0.6 in one of the two indicators but the other indicator was above 0.6. Finally, the questionnaire consisted of 42 items (Table 2).

### 3.4. Comprehensibility or Linguistic Validation Analysis

The reports on the items were minimal, with some spelling and grammatical changes and syntax structure. For example, experts requested reformulating item 13. The rest of the items were eliminated and were not reformulated. Items 2, 15, 40, 84, 93, and 95 were eliminated from the questionnaire because they presented a low level of comprehension with a value lower than 4. Item 13 was modified because it had values lower than 4 in comprehension; however, keeping it in the instrument was considered essential after its reformulation.

All the active experts who participated responded that they understood, without difficulty, the content of the final questionnaire designed, the concepts, and the answers to each item in terms of adequacy and comprehension. Finally, no expert detailed any doubts regarding the completion of the questionnaire.

## 4. Discussion

Given the importance of the increase in the prevalence and public health problem of chemsex consumption, we proposed at the beginning of the work the design and validation of a scale entitled “Chem-Sex Inventory” (CSI) to reach a consensus on the content in the development of a questionnaire [46,47], based on the Delphi method and analyzing the content validity of the questionnaire to be able to know the behaviors that predict consumption, allowing a better approach to people who practice chemsex and an advance in research on this type of addiction [48].

As a strength of the study, this instrument will facilitate the development of future studies to analyze and relate its construct to different variables such as substance use, impulsivity, altered body perception, risk of psychosis, risk of suicide, anxiety, and depression. Its application will allow multidisciplinary teams of professionals to plan and develop a better approach to chemsex patients and knowledge of their behavior.

The Delphi method has been shown to be relevant and helpful in health research [49] to obtain reasoned, consensual, and individualized opinions concerning the analysis and reflection on a given research objective [50,51].

First, professionals who had practice-based knowledge and experience in chemsex and could make valid contributions to the study were chosen as experts. Validation aims to ensure that the questionnaire documents what it is intended to measure and that its design and validation are of rigorous scientific quality. Such validation was carried out with experts to achieve an optimal level of validity, defined as the degree to which all representative indicators intended to be assessed are included in the questionnaire [48].

Furthermore, the group of experts consisted of a heterogeneous group from different regions and work services, which is fundamental because it allowed a different view from other points of view. Various studies indicate the strengths of using the modified Delphi technique as each round is conducted anonymously and independently, with the advantage of opportunity for participation in the study and reducing response bias that can appear in group settings [42]. Thus, all expert group participants had equal opportunity to participate in the study, reducing the risk of response bias that can arise in group settings [42,52].

Secondly, the methodology used was the Delphi technique, a consensus technique that allows quantitative estimators to be obtained through the degree of agreement among the participants. This is an effective method for building and creating consensus in a group without the group of experts having to meet in person [47], but rather contacting each of the group members via e-mail. With all of the above, it is necessary to have validated tools adapted to our environment that allow us to know the behavior and conduct of people who practice chemsex.

Regarding the characteristics of the present study, we can assure that the content validity is high. As we have seen, the decision-making of the group of experts has become increasingly uniform in the different Delphi stages, which is reflected in the significant increase in the congruence index of the items included [48]. The group of experts considered that the 42 items of the “Chem-Sex Inventory” (CSI) questionnaire, having a high level of content validity, capture aspects related to the predictive behavior of chemsex and therefore present a high level of content validity [42]. Being the first version of the questionnaire, the research group eliminated second-round items with a CVI and Aiken’s V higher than 0.6 as they were not relevant to assess the risk behaviors of the chemsex phenomenon and kept items with a CVI and Aiken’s V lower than 0.6, which were considered relevant to the study.

The Delphi method showed a high degree of agreement among experts in evaluating the questionnaire [48]. In the absence of instruments to assess the risk factors of the chemsex phenomenon in the literature reviewed, developing new tools is necessary [53]. This study can be used to inform other researchers in their efforts to validate the content of behaviors predictive of the chemsex phenomenon [47]. Following this, researchers can conduct a study of the LGTBI+ population that performs chemsex to be able, after the resulting analysis, to assess additional aspects of its validity and reliability and, based on the results, perform item reduction and subsequently establish a cut-off point to discriminate risk and allow for group stratification.

In this sense, the panel of experts made a quantitative and qualitative contribution that allowed for improving the tool [54], obtaining very positive values in all dimensions and their assessment category, namely, the relevance of the reference question and its response category, and relevance to the object of research, clarity, adequacy and comprehension of the wording, structure, and sequence of dimensions and questions. After content validation by experts, a final questionnaire was developed, which is in the process of analyzing other psychometric properties.

For future studies, some limitations should be kept in mind: First, a diverse sample of experts working in different systems and autonomous communities was selected to obtain a broader perspective on the chemsex phenomenon. Although previous research identified snowball sampling as an effective method for identifying expert groups in Delphi studies [55], it was found that the application of this sampling method in the present study may have resulted in the inclusion of some non-experts in the sample.

This may indicate that those with less experience may have limited exposure to some aspects of chemsex practice. As this was a study based on expert opinion, and although the team of experts was sufficiently broad and knowledgeable about the phenomenon, there is always the possibility that not all aspects or dimensions of the phenomenon were addressed. Another limitation is that the resulting questionnaire is relatively long, in our opinion, which may lead to a lack of responses in future research. Since this is the first version of the “Chem-Sex Inventory” (CSI) questionnaire, the research team aims to shorten it in successive versions while maintaining its properties. Therefore, future research using expert samples should consider how participants’ experiences fit the research objectives.

Once the instrument has been constructed, a subsequent pilot test is planned to analyze the psychometric properties of the “Chem-Sex Inventory” (CSI) questionnaire at the national level and a nationwide study of all the autonomous communities is planned to validate the instrument in men who have sex with men.

## 5. Conclusions

In conclusion, the results of the present study allow us to conclude that the questionnaire designed to determine the risk factors for the chemsex phenomenon has a high level of content validity and can, therefore, be used in the different emergency departments, primary care, psychiatry, and infectious disease departments for this purpose. It should be noted that although this instrument may be potentially helpful, other psychometric properties should be evaluated to ensure its validity and structure.

## Figures and Tables

**Table 1:** Demographic data of the panel of experts participating in the Delphi method.

Characteristics	N	%
Gender		
Male	21	(70)
Female	9	(30)
Position		
LGTBI+ Collective	4	(13.33)
Psychiatry Service	8	(26.66)
Primary Care	6	(20)
Urgent Care/Emergency Service	5	(16.66)
Infectious Diseases Service/STIs Clinic ^1^	7	(23.33)
Autonomous Community		
Aragon	1	(3.33)
Extremadura	2	(6.66)
Canary Islands	2	(6.66)
Catalonia	5	(16.66)
Valencian Community	1	(3.33)
La Rioja	3	(10)
Madrid	16	(53.33)
Profession		
Physician	10	(33.33)
Nurse	17	(56.66)
Psychologist	2	(6.66)
Occupational Therapist/Sexologist	1	(3.33)

^1^ STI (sexually transmitted infection).

**Table 2:** The content validity index (CVI), Aiken’s V, and the kappa for each item.

Item Number First Round	Items	CVI	^1^ k	Aiken’s V	^2^ IC CVI 95% (0.502–0.942)
1	1. Have you felt nervous, anxious or very upset?	0.900	0.900	0.900	(0.833–0.942)
4	2. Have you ever had difficulty relaxing?	0.900	0.900	0.850	(0.775–0.903)
6	3. Have you been easily annoyed or irritated?	0.933	0.933	0.875	(0.804–0.923)
7	4. Have you been afraid as if something terrible would happen?	0.733	0.732	0.767	(0.683–0.833)
8	5. Have you had little interest or pleasure in doing things?	0.833	0.833	0.817	(0.738–0.876)
9	6. Have you ever felt discouraged, depressed or hopeless?	0.867	0.867	0.867	(0.794–0.916)
11	7. Have you ever felt tired or low energy?	0.733	0.732	0.783	(0.701–0.848)
12	8. Have you had no appetite or, on the contrary, have you eaten too much?	0.667	0.657	0.725	(0.639–0.797)
13	9. Have you ever felt bad about yourself, felt that you are a failure or failing yourself?	0.800	0.800	0.817	(0.738–0.876)
14	10. Have you had difficulty concentrating on doing everyday things, such as reading the newspaper or watching television?	0.867	0.867	0.800	(0.720–0.862)
16	11. Have you ever had thoughts that you would be better off dead or harming yourself?	0.800	0.800	0.783	(0.701–0.848)
17	12. Do familiar environments sometimes seem strange, confusing, threatening or unreal to you?	0.767	0.766	0.733	(0.648–0.804)
21	13. Have you ever felt like you were not in control of your thoughts or ideas?	0.833	0.833	0.750	(0.666–0.819)
22	14. Have you ever had difficulty following your conversation because you ramble or lose concentration too much when talking?	0.800	0.800	0.742	(0.657–0.812)
24	15. Have you ever felt that other people are watching or talking about you?	0.800	0.800	0.767	(0.683–0.833)
25	16. Have you occasionally noticed any sensation on or under the skin, such as bugs?	0.600	0.565	0.658	(0.570–0.737)
28	17. Have you ever worried that something might go wrong in your mind?	0.667	0.657	0.717	(0.630–0.790)
30	18. Have you ever felt confused about whether something that happened to you was real or imaginary?	0.733	0.732	0.725	(0.639–0.797)
34	19. Have you ever had feelings of distrust towards other people?	0.800	0.800	0.758	(0.674–0.826)
35	20. Have you ever seen unusual things such as flashes, flames, glaring lights or geometric shapes?	0.733	0.732	0.692	(0.604–0.767)
36	21. Have you ever seen things that other people cannot see?	0.567	0.512	0.608	(0.519–0.691)
37	22. Do you feel people find it difficult to understand your words?	0.633	0.614	0.650	(0.561–0.729)
41	23. Have you ever had thoughts about your mind going faster than normal?	0.700	0.696	0.733	(0.648–0.804)
52	26. In the last few days, have you felt that you were acting impulsively?	0.867	0.867	0.783	(0.701–0.848)
66	30. Do you feel restless in classes or lectures if you have to listen to someone talk for a long time?	0.633	0.614	0.700	(0.613–0.775)
73	32. Have you ever had chest pains?	0.700	0.696	0.708	(0.622–0.782)
76	34. Have you ever felt your heart beating faster than usual?	0.800	0.800	0.800	(0.720–0.862)
77	35. Have you ever felt short of breath?	0.767	0.766	0.775	(0.692–0.841)
82	36. Have you had difficulty sleeping?	0.900	0.900	0.883	(0.814–0.929)
85	37. Have you ever really had the idea of committing suicide?	0.833	0.833	0.792	(0.711–0.855)
86	38. Have you thought about how you would carry it out?	0.800	0.800	0.775	(0.692–0.841)
87	39. Have you ever tried to take your own life?	0.700	0.696	0.700	(0.613–0.775)
88	40. Have you ever felt that you have difficulty maintaining your attention, are easily distracted, or cannot concentrate?	0.667	0.657	0.683	(0.596–0.760)
92	42. Do you consider yourself a person with anger attacks that are difficult to predict?	0.867	0.867	0.783	(0.701–0.848)
97	43. At any time, have you considered yourself to be a person who changes moods frequently?	0.833	0.833	0.767	(0.683–0.833)
100	45. Have you ever felt people were dropping hints or saying things with a double meaning?	0.667	0.657	0.675	(0.587–0.752)
104	46. Have you ever felt that people look at you strangely because of your appearance?	0.633	0.614	0.650	(0.561–0.729)
106	47. Have you ever felt your thoughts were being pulled out of your head?	0.600	0.565	0.617	(0.527–0.699)
107	48. Have you ever felt your thoughts were not your own?	0.733	0.732	0.708	(0.622–0.782)
109	49. Have you ever felt your thoughts constantly repeated in your mind?	0.700	0.696	0.717	(0.630–0.790)
110	50. Have you ever felt as if you were under the control of any external force or power?	0.600	0.565	0.592	(0.502–0.675)
112	51. Have you ever heard voices when you were alone?	0.733	0.732	0.717	(0.630–0.790)

^1^ K (kappa); ^2^ IC (confidence interval). Note: The CSI questionnaire was designed in Spanish; this table shows items translated into English (but not an English-validated version).

## Data Availability

The data that support the findings of this study are available from the corresponding authors upon reasonable request.

## Appendix A

**Table A1:** Index of content validity and comprehension of each item in the Delphi procedure in the first round.

Items	Relevant to the Topic		Understanding of the Topic
	CVI	Aiken’s V	Average Score
1. Have you ever felt nervous, anxious or very upset?	0.946	0.903	4.39
2. Have you not been able to stop worrying?	0.676	0.736	3.39
3. Have you worried too much about different things?	0.622	0.681	3.56
4. Have you had difficulty relaxing?	0.919	0.840	4.64
5. Have you ever felt so restless that you could not sit still?	0.568	0.667	3.50
6. Have you been easily annoyed or irritated?	0.919	0.903	4.61
7. Have you been afraid as if something terrible would happen?	0.811	0.792	4.22
8. Have you had little interest or pleasure in doing things?	0.838	0.847	4.39
9. Have you ever felt discouraged, depressed or hopeless?	0.865	0.861	4.53
10. Have you had difficulty falling, staying, or sleeping too much?	0.865	0.903	4.47
11. Have you ever felt tired or low on energy?	0.865	0.840	4.47
12. Have you ever felt lacking in appetite or, on the contrary, have you eaten too much?	0.703	0.757	4.19
13. Have you ever felt bad about yourself or that you are a failure or failing yourself?	0.865	0.826	3.44
14. Have you had difficulty concentrating, such as reading the newspaper or watching television?	0.784	0.799	4.17
15. Have you ever moved or talked so slowly that other people around you have noticed or, conversely, when you are nervous or restless do you move more than usual?	0.595	0.681	3.39
16. Have you ever had thoughts that you might be better off dead or hurting yourself?	0.919	0.889	4.28
17. Do familiar environments sometimes seem strange, confusing, threatening or unreal to you?	0.784	0.792	4.25
18. Have you ever heard unusual sounds such as popping, hissing, clapping, or ringing in your ears?	0.676	0.729	4.06
19. Do the things you see seem different from how they usually are (brighter or duller, bigger or smaller, or changed in some other way?	0.649	0.701	4.00
20. Have you had any experiences of telepathy, seer or fortune teller powers?	0.405	0.597	3.64
21. Have you ever felt like you were not in control of your thoughts or ideas?	0.838	0.826	4.33
22. Have you had difficulty following your topic because you ramble or lose track when speaking?	0.892	0.819	3.78
23. Have you ever had a strong feeling or belief that you possess any kind of unusual gifts or talents, for example, telepathy?	0.514	0.604	3.81
24. Have you ever felt that other people are watching or talking about you?	0.892	0.875	4.67
25. Have you ever noticed any sensation on or under the skin, such as bugs?	0.757	0.806	4.19
26. have you ever felt suddenly distracted by distant sounds you were unaware of?	0.568	0.701	3.89
27. Have you ever felt that there was a person or force around you, even though you could not see anyone?	0.676	0.694	4.06
28. Have you ever worried that something might go wrong in your mind?	0.865	0.861	4.28
29. Have you ever felt that you did not exist, that the world did not exist or that you were dead?	0.568	0.653	3.83
30. Have you ever felt confused about whether something that happened to you was real or imaginary?	0.811	0.806	4.31
31. Have you ever held beliefs others would find difficult to believe?	0.649	0.708	3.75
32. Have you felt that parts of your body have changed in any way, or that parts of your body were functioning differently?	0.622	0.715	3.92
33. Have you felt that your thoughts are sometimes so intense that you can almost hear them?	0.676	0.722	4.14
34. Have you ever had feelings of distrust towards other people?	0.811	0.840	4.47
35. Have you ever seen unusual things such as flashes, flames, glaring lights, or geometric shapes?	0.757	0.736	4.17
36. Have you ever seen things that other people cannot see?	0.757	0.764	4.08
37. Do people sometimes have difficulty understanding what you are saying?	0.757	0.771	4.08
38. Have you planned your tasks carefully?	0.459	0.590	3.69
39. Have you ever done things without thinking about them?	0.595	0.694	4.17
40. Have you hardly ever taken things to heart or are you not easily disturbed?	0.378	0.549	2.50
41. Have you ever thought your mind is going faster than normal?	0.811	0.819	4.14
42. Do you consider yourself a person who plans your trips?	0.270	0.438	4.08
43. Do you consider yourself a person with self-control?	0.811	0.785	4.25
44. Are you a person who can concentrate easily?	0.730	0.715	4.39
45. Are you a person who saves regularly?	0.514	0.604	4.42
46. Do you find it difficult to sit still for long periods?	0.784	0.764	4.39
47. Do you think things through carefully?	0.568	0.653	4.08
48. Do you plan to have a steady job or strive to ensure that you will have money to pay your expenses?	0.514	0.625	3.86
49. Do you ever say things without thinking them through?	0.595	0.681	4.19
50. Do you like to think about complicated problems?	0.459	0.576	3.94
51. Do you change jobs frequently?	0.514	0.660	4.28
52. Do you act impulsively?	0.865	0.840	4.28
53. Do you get bored easily trying to solve problems in your mind?	0.514	0.653	3.94
54. Do you visit the doctor and dentist frequently?	0.514	0.632	4.00
55. Do you do things as they occur to you?	0.595	0.681	4.06
56. Do you consider yourself a person who thinks without being distracted, that is, can you focus your mind on one thing for a long time?	0.622	0.694	4.08
57. Do you change housing frequently, i.e., move frequently or dislike living in the same place for a long time?	0.378	0.542	4.00
58. Do you buy things on impulse?	0.595	0.694	4.25
59. Do you complete all the activities you start?	0.622	0.688	4.33
60. Do you walk and move quickly?	0.324	0.576	4.11
61. Do you solve problems by experimenting, i.e., do you solve problems by trying a possible solution and seeing if it works?	0.541	0.667	3.83
62. Do you spend more in cash or credit than you earn?	0.622	0.715	3.92
63. Have you noticed that you talk faster than usual?	0.649	0.708	3.81
64. Have you ever had strange thoughts when you were thinking?	0.676	0.736	3.61
65. Are you more interested in the present than in the future?	0.622	0.688	4.33
66. Do you feel restless in classes or lectures if you have to listen to someone talk for a long period?	0.595	0.667	4.17
67. Do you plan for the future, i.e., are you more interested in the future than in the present?	0.595	0.632	4.00
68. Have you ever had stomach pain?	0.459	0.576	4.50
69. Have you ever had back pain?	0.351	0.528	4.44
70. Have you ever had pain in your arms, legs or joints (knees, hips)?	0.432	0.563	4.28
71. Have you had menstrual cramps or other problems with your periods?	0.297	0.458	3.97
72. Have you ever had headaches?	0.541	0.646	4.58
73. Have you ever had chest pains?	0.676	0.688	4.47
74. Have you ever had dizziness?	0.703	0.701	4.39
75. Have you had any episodes of fainting?	0.649	0.694	4.39
76. Have you ever felt your heart beating faster and faster?	0.811	0.806	4.44
77. Have you ever felt short of breath?	0.865	0.799	4.44
78. At any time have you experienced pain or problems during sexual penetration?	0.649	0.708	4.28
79. Have you had episodes of constipation or diarrhea?	0.514	0.632	4.47
80. Have you ever had nausea?	0.622	0.681	4.50
81. Have you ever felt tired or low on energy?	0.865	0.847	4.58
82. Have you had difficulty sleeping?	0.946	0.903	4.75
83. Have you ever felt that life was not worth living?	0.919	0.875	4.67
84. Have you ever wished you were dead or could sleep and wake up?	0.784	0.771	3.08
85. Have you ever really had the idea of committing suicide?	0.865	0.861	4.22
86. Have you thought about how you would do it?	0.811	0.806	4.36
87. Have you ever tried to take your own life?	0.811	0.847	4.61
88. Have you ever felt that you have difficulty maintaining your attention, are easily distracted, unable to concentrate?	0.838	0.833	4.42
89. Have you ever felt that you are impulsive, impatient, poorly tolerate pain or frustration?	0.757	0.785	3.78
90. Do you ever feel uncooperative, uncaring, and demanding of yourself?	0.541	0.694	3.69
91. Do you consider that you are violent or threatening to people?	0.649	0.750	4.28
92. Do you consider yourself an explosive person or a person with anger attacks that are difficult to predict?	0.757	0.785	4.39
93. Do you consider yourself restless, rubbing, moaning or other self-stimulating behavior?	0.432	0.611	2.86
94. Do you consider yourself restless, come and go, and move around excessively?	0.649	0.729	3.72
95. Do you consider yourself a person who exhibits repetitive, motor or verbal behaviors?	0.541	0.688	3.44
96. Do you consider yourself a fast or excessive talker?	0.595	0.722	4.17
97. Do you consider yourself a person who changes mood frequently?	0.892	0.840	4.50
98. Do you consider yourself a person who cries or laughs easily and excessively?	0.757	0.750	4.08
99. Do you consider yourself a person who insults or hurts others?	0.541	0.667	3.69
100. Have you ever felt people were dropping hints or making double entendres?	0.757	0.757	4.00
101. Have you ever felt some people are not what they appear to be?	0.649	0.708	3.83
102. Have you ever felt that you are being persecuted in any way?	0.730	0.771	4.25
103. Have you ever felt as if there was a conspiracy against you?	0.703	0.764	4.19
104. Have you ever felt that people look at you strangely because of your appearance?	0.811	0.771	4.33
105. Have you felt that certain electronic devices, such as computers, phones or tablets, can influence your thinking?	0.676	0.715	4.19
106. Have you felt your thoughts were being pulled out of your head?	0.676	0.708	3.94
107. Have you ever felt as if your thoughts were not your own?	0.811	0.771	4.36
108. Have you ever had intense thoughts that you worried others might hear them?	0.595	0.688	4.22
109. Have you ever felt your thoughts constantly repeated in your mind?	0.811	0.778	4.39
110. Have you ever felt as if you were under the control of any external force or power?	0.649	0.694	4.11
111. Have you ever felt like a look-alike had impersonated a family member, friend or acquaintance?	0.459	0.597	3.94
112. Have you ever heard voices when you were alone?	0.784	0.778	4.42
113. Have you ever heard voices talking to each other when you were alone?	0.703	0.722	3.89
114. Have you ever seen objects, people or animals that others could not see?	0.622	0.688	4.17
